# Epoxy/Ionic Liquid-Modified Mica Nanocomposites: Network Formation–Network Degradation Correlation

**DOI:** 10.3390/nano11081990

**Published:** 2021-08-03

**Authors:** Maryam Jouyandeh, Vahideh Akbari, Seyed Mohammad Reza Paran, Sébastien Livi, Luanda Lins, Henri Vahabi, Mohammad Reza Saeb

**Affiliations:** 1 Université de Lorraine, CentraleSupélec, LMOPS, F-57000 Metz, France; maryam.jouyande@gmail.com (M.J.); vahidehakbari1991@gmail.com (V.A.); 2 Center of Excellence in Electrochemistry, School of Chemistry, College of Science, University of Tehran, Tehran 1417935840, Iran; smrparan@gmail.com; 3 Université de Lyon, CNRS, UMR 5223, Ingénierie des Matériaux Polymères, INSA Lyon, F-69621 Villeurbanne, France; sebastien.livi@insa-lyon.fr; 4 Université de Lyon, CNRS, UMR 5510, MATEIS, INSA Lyon, F-69621 Villeurbanne, France; luandaqmc@gmail.com; 5 Department of Polymer Technology, Faculty of Chemistry, Gdańsk University of Technology, G. Narutowicza 11/12, 80-233 Gdańsk, Poland

**Keywords:** cure index, cure kinetics, epoxy nanocomposite, mica, ionic liquids, degradation kinetics

## Abstract

We synthesized pristine mica (Mica) and N-octadecyl-N’-octadecyl imidazolium iodide (IM) modified mica (Mica-IM), characterized it, and applied it at 0.1–5.0 wt.% loading to prepare epoxy nanocomposites. Dynamic differential scanning calorimetry (DSC) was carried out for the analysis of the cure potential and kinetics of epoxy/Mica and epoxy/Mica-IM curing reaction with amine curing agents at low loading of 0.1 wt.% to avoid particle aggregation. The dimensionless *Cure Index* (*CI*) was used for qualitative analysis of epoxy crosslinking in the presence of Mica and Mica-IM, while qualitative cure behavior and kinetics were studied by using isoconversional methods. The results indicated that both Mica and Mica-IM improved the curability of epoxy system from a *Poor* to *Good* state when varying the heating rate in the interval of 5–15 °C min^−1^. The isoconversional methods suggested a lower activation energy for epoxy nanocomposites with respect to the blank epoxy; thus, Mica and Mica-IM improved crosslinking of epoxy. The higher order of autocatalytic reaction for epoxy/Mica-IM was indicative of the role of liquid crystals in the epoxide ring opening. The glass transition temperature for nanocomposites containing Mica and Mica-IM was also lower than the neat epoxy. This means that nanoparticles participated the reaction because of being reactive, which decelerated segmental motion of the epoxy chains. The kinetics of the thermal decomposition were evaluated for the neat and mica incorporated epoxy nanocomposites epoxy with varying Mica and Mica-IM amounts in the system (0.5, 2.0 and 5.0 wt.%) and heating rates. The epoxy/Mica-IM at 2.0 wt.% of nanoparticle showed the highest thermal stability, featured by the maximum value of activation energy devoted to the assigned system. The kinetics of the network formation and network degradation were correlated to demonstrate how molecular-level transformations can be viewed semi-experimentally.

## 1. Introduction

Epoxy resin is the main material in diverse fields of technology for developing painting materials, electronic devices, adhesives, surface coatings, automotive exterior parts, shipbuilding and aerospace; thus, mechanical and thermal properties of epoxy have been widely studied [1,2,3,4]. Epoxy nanocomposites have also been in the center of attention and their thermal, corrosion protection, mechanical, and flame retardant properties have been the subject of many works [5,6,7,8,9]. The desired properties of thermoset nanocomposites are strongly related to the nanoparticles [10,11,12,13]. Properties of the thermoset polymer composites are dependent on the crosslinking reactions, as well as the size, the shape and the surface functionality of the nanoparticles [14,15,16]. In this regard, appropriate selection of nanoparticles can guarantee the required properties [17,18,19].

**Figure 1:**
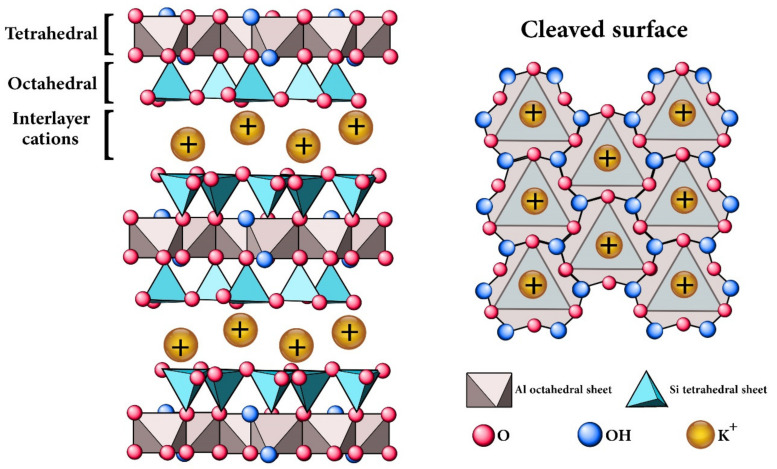
The schematic structure of mica mineral with lamellar structure.

There are quite a wide range of synthetic and natural nanoparticles for the modification of polymers, depending on the performance [20,21], among which clay nanoplatelets have been frequently chosen because of their low cost, abundance, and non-toxicity [22,23,24,25]. Mica is a natural silicate mineral with a lamellar structure representing acceptable thermal and chemical stability and available in large quantity and low cost (Figure 1). Mica can enhance the mechanical properties of polymer composites, due to its multi-layer structure that supports enlarged infiltration path of corrosive medium and prevents small molecules from penetrating into the polymer network [26,27].

Many publications have addressed properties of epoxy composites containing mica nanoclay. The presence of mica in epoxy composites delays thermoelectric aging of epoxy insulation and improves the thermal properties of the epoxy [28]. Moreover, some reports suggest that mica incorporation into epoxy adhesive enhances the compressive and bending strength [29,30,31,32,33]. Nevertheless, the presence of mica makes the crosslinking kinetics of the epoxy indeed complex and thereof affects the gelation behavior of epoxy resins in insulation usage. As a consequence of such changes, incorporation of mica decelerates the kinetics of the crosslinking reaction [34]. Therefore, mica should be modified to attain acceptable crosslinking in epoxy systems. Some used hybrid systems based on mica—either physically or chemically—and other nanoparticles or blend of epoxy with other polymers to compensate for poor epoxy-mica curing reaction [35]. For instance, combined use of mica and multi-walled carbon nanotubes revealed a better anti-corrosion properties compared to the mica only [27]. However, to the best of our knowledge, no report is available on curing kinetics and crosslinking reaction of epoxy/mica nanocomposites. Overall, mica can affect the curing process and increases the glass transition temperature (*T_g_*) of the system according to isothermal investigations. However, reports solely address the degree of conversion [26]. Therefore, a detailed kinetics study is essential to explore structure–property relationships in epoxy/mica nanocomposites.

In the light of above, exploring the correlation between network formation and network degradation is of crucial importance from a practical point of view. In the present work, highly intercalated mica based on imidazolium which is an ionic liquid (IM) was employed for the sake of appropriate dispersion throughout the epoxy resin. Cure kinetics analyses were carried out applying nonisothermal differential scanning calorimetry (DSC) with a varying heating cycle. The effects of pristine mica and mica-IM, hereafter referred to as Mica and Mica-IM, on crosslinking kinetics of epoxy were thereby compared. The dimensionless *Cure Index* (*CI*) was employed for the qualitative analysis of the state of cure of the systems, while detailed kinetics analysis was performed applying isoconversional approaches, to name Kissinger–Akahira–Sunose (*KAS*) and *Friedman*. Accordingly, we visualized the contributions of autocatalytic and non-catalytic reactions to epoxy crosslinking in the presence of Mica and Mica-IM. Dynamic mechanical analysis (DMA) was also applied, which allowed for taking the signature of viscoelastic behavior of epoxy nanocomposites. Finally, thermal decomposition mechanism of composites was characterized by the aid of thermogravimetric analysis (TGA).

## 2. Materials and Methods

The materials used together with the methodology applied in the synthesis of imidazolium ionic liquid and its organic modification, prepation of epoxy nanocomposites and characetrization of nanocomposites for cure kinetics and DMA together with TGA are provided in detail in the Supplementary Materials (SM), Section S1.

## 3. Results and Discussion

### 3.1. Microstructural Analyses

**Figure 2:**
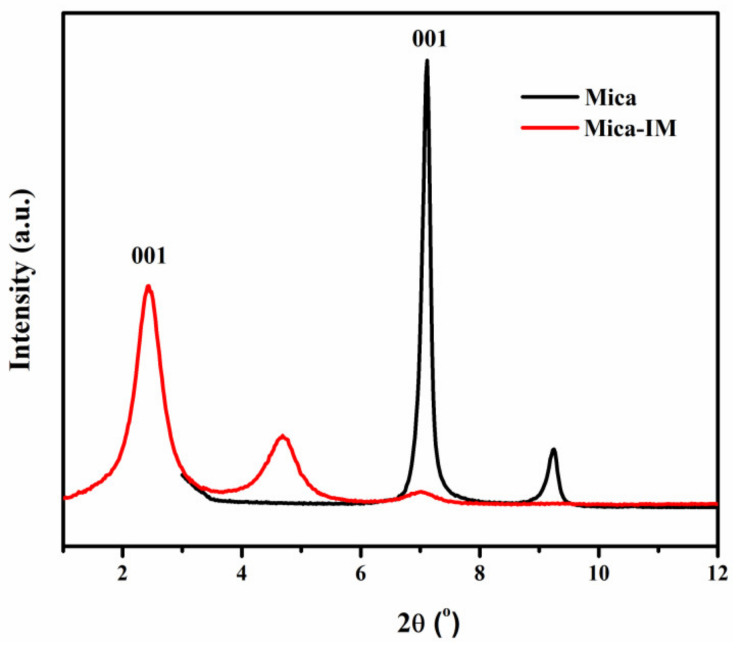
X-ray diffraction spectrum of the Mica-IM.

We used X-ray diffraction (XRD), in order to confirm surface treatments of mica with imidazolium salt (formation of Mica-IM). As shown in Figure 2, before surface modification by ionic liquid, we could see the basal spacing peak of the pristine mica at around 1.2 nm. The corresponding (001) diffraction peak was sharply appeared at 2θ of 7.11°, more or less in the order of d-spacing reported for the virgin ME-100 [36,37]. After modification of mica by the use of cation exchange reaction, we can see that the diffraction peak of (001) was significantly moved towards an obviously lower 2θ of about 2.40°, which is identical to an interlayer distance in the order of 3.7 nm. Thus, it can be concluded that a paraffinic conformation is the case, and alkyl chains are at trans-trans configuration [36,38,39]. In other words, large galleries are formed corresponding to the intercalated morphology formed in the system. It can be speculated that Mica-IM could be more appropriately dispersed throughout the epoxy resin than that of Mica.

**Figure 3:**
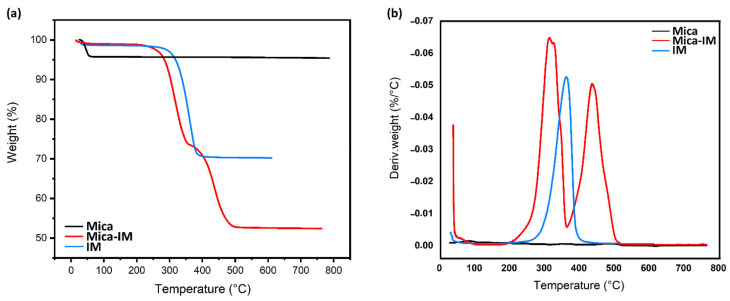
Typical of variation of weight loss, TGA (**a**) and derivative of weight loss, DTG (**b**) of the studied samples vs. temperature (heating rate: 20 °C min^−1^, atmosphere: nitrogen).

Based on previous studies [40], the proportion of imidazolium ionic liquid that chemically or physically linked between mica layers was recognized through thermogravimetric analysis (TGA), as seen in Figure 3.

Figure 3 compares the thermal weight loss behavior of Mica, IM and Mica-IM, where a very different behavior can be seen. Mica remains almost stable even up to 800 °C, while two-stage degradation of Mica-IM takes place at 309 and 431 °C, respectively. The initial degradation stage is signaled by a peak correlated with the inherent thermal stability of imidazolium ionic liquid, possibly species physically absorbed onto the surface of mica as also can be seen in TGA of IM. The second decomposition stage is featured by the peak corresponding to the enlargement of clay galleries. The neat imidazolium ionic liquid functionalized with different alkyl chain lengths is found to have a maximum degradation around 300–320 °C. In addition, several washes of ionic liquid-modified layered silicates with water as well as organic solvents such as methyl alcohol give reason for recognition of physically adsorbed and/or intercalated species. Thus, the dispersant effect of the physisorbed ionic liquid on nanoparticles is confirmed [41].

**Figure 4:**
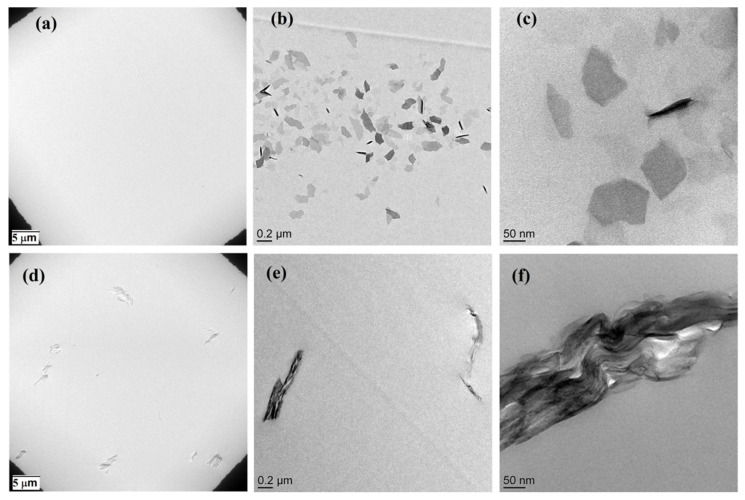
TEM images of the Mica-IM (**a**–**c**) and Mica (**d**–**f**) dispersed into epoxy matrix at 0.1 wt %.

Although modification of nanoparticles was confirmed through the XRD method, TEM gives a close-up of the morphology of mica in the epoxy nanocomposites. In Figure 4d–f, the agglomeration of raw mica in the epoxy matrix can be obviously seen. By contrast, the dispersion of modified nanofiller in the matrix is clear on the same scale. By looking at images provided from higher magnification, the inability of raw mica nanoparticles to disperse in the epoxy resin can be clearly observed, while the role of imidazolium-based ionic liquid in dispersion of mica is clearly demonstrated (Figure 4a–c). These results were confirmed by the XRD spectra that indicated the successful intercalation and modification of Mica by the ionic liquid. The higher basal spacing and the presence of functional gropes on the surface of Mica-IM resulted in excellent dispersion of the modified Mica through the epoxy resin.

**Figure 5:**
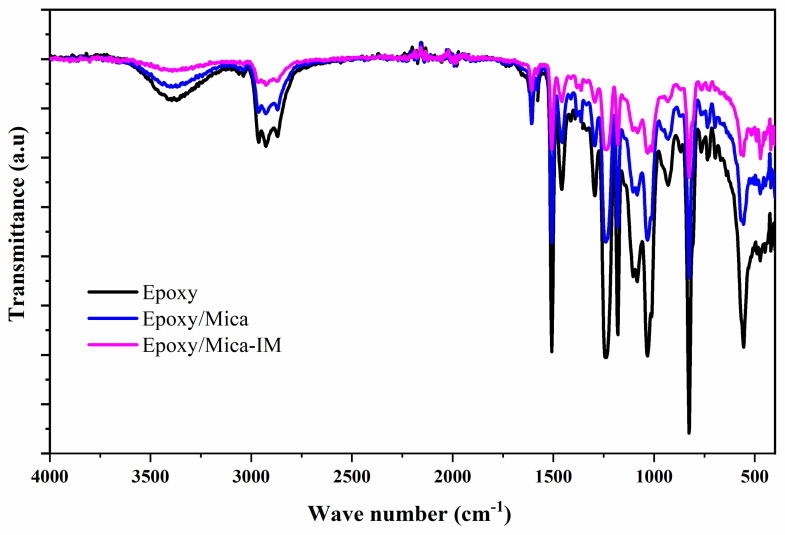
FTIR spectra obtained for the studied samples, blank epoxy and epoxy containing 0.1 wt % Mica or Mica–IM.

We compared the FTIR spectra of neat epoxy with epoxy nanocomposites containing Mica and Mica-IM to evaluate the changes in chemical functionality, as shown in Figure 5. At 3394 cm^−1^ we can see a broad peak, indicative of stretching vibration bonds present in hydroxyl groups. Moreover, symmetrical stretching methyl group (CH_3_) bonds appeared at 2920 cm^−1^, while asymmetrical ones formed at 2870 cm^−1^. The stretching vibration and absorption of the ring were also detected at 825 and 1510 cm^−1^, respectively. At around 1030 and 1234 cm^−1^ we can see two explicit adsorptions assigned to C–O–C and C–N stretching, one-to-one. The absorption bands in the 1610 cm^−1^ indicate C=C bond of aromatic rings can be seen for EP/Mica and EP/Mica-IM [42,43]. The vibration of C–N band present in imidazolium ionic liquid appeared at 1380 cm^−1^ for EP/Mica-IM sample [44]. Evidently, the broad OH peak with obvious intensity at 3394 cm^−1^ decreased for EP/Mica and EP/Mica-IM due to the interaction during epoxy ring opening [45].

### 3.2. Cure Analysis

**Figure 6:**
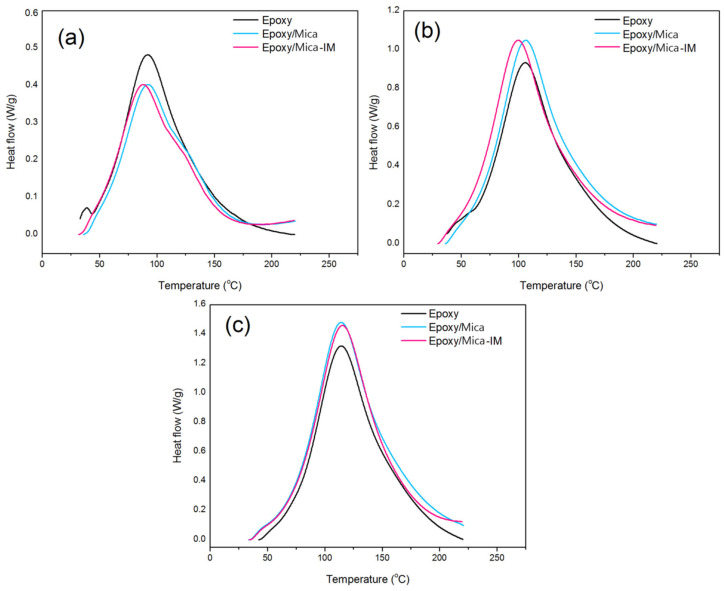
Nonisothermal thermograms of DSC conducted on the blank epoxy and epoxy systems with 0.1 wt % Mica or Mica-IM nanoparticles, varying β as 5 °C min^−1^ (**a**), 10 °C min^−1^ (**b**), and 15 °C min^−1^ (**c**).

Nonisothermal cure analysis was performed varying the heating rates in DSC for the blank epoxy resin and also epoxy with 0.1 wt % Mica or Mica-IM, Figure 6. The single exothermic peaks unconditionally observed in thermograms approves single-step curing kinetic assumption [46].

#### 3.2.1. Qualitative Cure Analysis

To qualitatively analyze curing we used *Cure Index* (*CI*) [47]. Equation (1) defines the calculation of *CI* values:

(1) $$CI=\Delta H^{*}\times \Delta T^{*}$$

Equations (2) and (3) can be used to calculate the quantities of ΔH* and ΔT*, respectively:

(2) ΔH∗=ΔHCΔHRef

(3) ΔT∗=ΔTCΔTRef

Δ*H_Ref_* and Δ*H_C_* in these formula are the total values of enthalpy of cure dedicated to the neat epoxy and epoxy nanocomposites, respectively. Furthermore, the cure temperature ranges for EP/Mica, EP/Mica-IM and EP are appeared into Δ*T_C_* and Δ*T_Ref_*, respectively. Detailed analysis is available in Table 1.

Table 1 suggests that increasing *β* from 5 to 15 °C min^−1^ increased both *T_P_* and Δ*H*, as expected from previous investigations [48]. The presence of Mica and Mica-IM in the epoxy nanocomposites causes a reduction in the *T_onset_*, which is a signature of the facilitation of the cure, except for the first rate of heating, 5 °C min^−1^. The same behavior was observed for the epoxy systems containing Mica-IM. The facilitated cure of epoxy caused by mica can be confirmed by higher Δ*H*_∞_ values obtained for epoxy nanocomposites whatever the heating rate. Nevertheless, for both Ep/Mica and Ep/Mica-IM systems cured at 15 °C min^−1^ the system was highly energetic and chains have no adequate time for interaction with Mica or Mica-IM with epoxy, as previously seen for different nanoparticles [49,50]. Correspondingly, the values of Δ*H*_∞_ for the assigned nanocomposites at 15 °C min^−1^ are lower compared to 10 °C min^−1^, but still higher than that for the reference epoxy. Similar results were reported in our previous works varying the heating rate [51,52].

**Figure 7:**
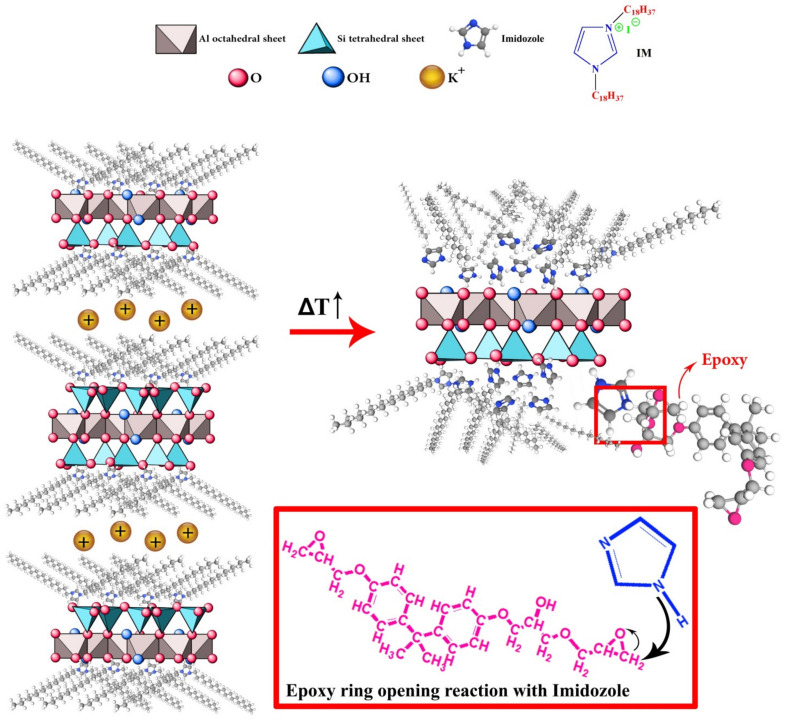
Schematic of interaction between the functionalized Mica (Mica-IM) and epoxy resin.

At the low heating rate the presence of Mica and Mica-IM decreased Δ*H*_∞_ values. However, at higher heating rates more kinetic energy was imposed to the system, such that the surface chemistry of Mica and Mica-IM contributed to the epoxy ring opening, reflected in Δ*H*_∞_ (Figure 7). Moreover, nano-size Mica and Mica-IM can diffuse into the epoxy crosslinked network in the diffusion-controlled zone (vitrification) and leads to occurrence of curing reaction in a wider temperature range, Δ*T* [53]. Through the vitrification, crosslink density of EP/Mica and EP/Mica-IM was increased [54].

Based on the CI values given in Table 1, the cure state of epoxy can be defined as the *Poor* ($CI<\Delta T^{*}$), *Good* ($CI>\Delta H^{*}$) and *Excellent* ($\Delta T^{*}<CI<\Delta H^{*}$) cases [55,56]. According to Table 1, addition of Mica and Mica-IM into the epoxy system and curing in DSC at 5 °C min^−1^ led to the *Poor* cure state, which is probably due to the insufficient energy available in the system which could energize curing moieties to be crosslinked [57,58]. Once heating rates increased to 10 and subsequently to 15 °C min^−1^, the Mica and Mica-IM were cured in a wider temperature interval and released more heat, resulting in a shift from the *Poor* to *Good* state [59].

#### 3.2.2. Quantitative Cure Analysis

The conversion of cure reaction, α can be simply obtained at each temperature by dividing Δ*H_T_* by Δ*H*_∞_ [60]:

(4) $$\alpha =\frac{\Delta H_{T}}{\Delta H_{\infty }}$$

**Figure 8:**
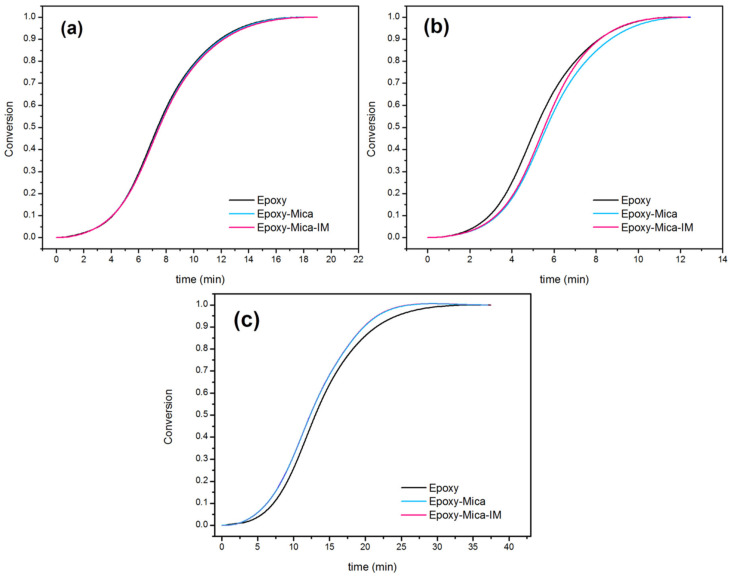
Conversion of cure reaction against time at *β* of 5 °C min^−1^ (**a**), 10 °C min^−1^ (**b**) and 15 °C min^−1^ (**c**) for blank epoxy and epoxy nanocomposites filled with 0.1 wt % Mica and Mica-IM.

The sigmoidal shape of the curves in Figure 8 is a signature of dominance of the autocatalytic epoxy crosslinking. The *α* values increases gradually at the early times of the curing process, while the slope of curves rose significantly in the middle of the cure reaction due to the occurrence of the gelation. Eventually, the cure is decelerated at late stages by leveling the curves off [61]. When cured slowly (*β* = 5 °C min^−1^), Mica and Mica-IM could not easily diffuse in the epoxy dense network, because of the lack of required energy. Therefore, the effect of nanoparticles was not pronounced at the low heating rate and resulted in the same curing rate with the neat epoxy. However, both Mica and Mica-IM played the role of cure promoter after vitrification (*β* = 10 °C min^−1^). Because of higher kinetic energy and adequate time in the system at 10 °C min^−1^, Mica and Mica-IM could possibly diffuse in the in epoxy network and stay until they react [62]. At the high heating rate (*β* = 15 °C min^−1^), the curing moieties had a shorter time for reaction, such that nanoparticles did not accelerate the curing reaction [63].

Kinetic data of epoxy nanocomposites are derived from the nonisothermal DSC based on isoconversional method. To achieve the amount of activation energy (*E_α_*) two different approaches were considered; *Friedman* as differential approach in contrast to Kissinger–Akahira–Sunose (*KAS*) representative of integral methods [64].

In each conversion rate, the reaction rate is only a function of temperature, with a reference on the isoconversional method assumption, which is calculated in Equation (5).

(5) $$\frac{d\alpha }{dt}=k(T)f(\alpha )$$

In the above equation, *f*(α) and *k*(*T*) are the reaction model and reaction rate constant, respectively, where:

(6) $$k(T)=A\exp(-\frac{E_{\alpha }}{RT})$$

In Equation (6), *R*, *T* and *A* are represented universal gas constant and absolute temperature and frequency factor, respectively. The following equation as a curing rate is derived from replacing Equation (6) in Equation (5):

(7) $$\frac{d\alpha }{dt}=A\exp(-\frac{E_{\alpha }}{RT})f(\alpha )$$

**Figure 9:**
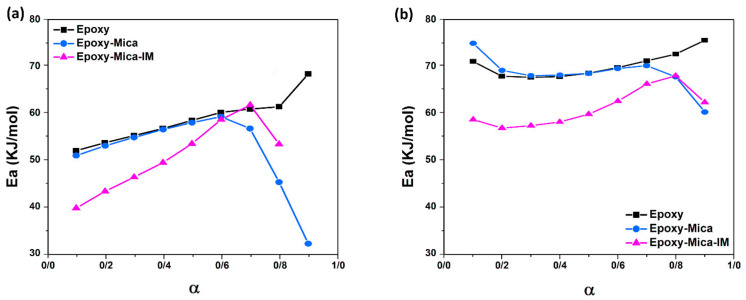
Change in Eα in terms of conversion based on *Friedman* model (**a**) and *KAS* model (**b**), for the blank epoxy and its nanocomposites.

According to the assumptions mentioned above, the differential *Friedman* and *KAS* methods for isoconversional model are described as Equations (S1) and (S2) in Supplementary Materials, respectively. The values of activation energy were obtained from *Friedman* and *KAS* methods as mentioned in Supplementary Materials Section S2. Figure 9a,b shows the activation energy derived from the *Friedman* and *KAS* models as a function of *α*, respectively. All plots follow an almost ascending trend until the middle of the reaction. This might be related to the shift in crosslinking state from chemical- to diffusion-control mechanism. The hindrance effect imposed by Mica and probably its improper dispersion into the epoxy matrix leads to obvious decline in activation energy in comparison with neat epoxy. However, the modification of nanoparticles with imidazolium is facilitated cure reaction and led to decrease the activation energy. Furthermore, significant drop in EP/Mica and EP/Mica-IM curves at higher conversion are because of possible esterification reaction between epoxy and hydroxyl groups [65].

The autocatalytic and non-catalytic mechanisms are studied by the use of *Freidman* and *Malek* methods as described by Equations (S3) and (S4 and S5) in Supplementary Materials, respectively.

The *Friedman* method approves having a nth-order cure mechanism, Figure S2, with a peak in *α* ranging in the interval 0.2–0.4, characteristic of autocatalytic crosslinking.

According to the *Malek* method (Figure S3), the z(*α*) plots obviously shows a maximum by *α_p_^∞^* in the same range. However, y(*α*) for *α_m_* has peaked around *α* equal to 0.1, demonstrating nth-order non-catalytic mechanism. Thus, an autocatalytic reaction model can explain curing whatever the system [66].

**Figure 10:**
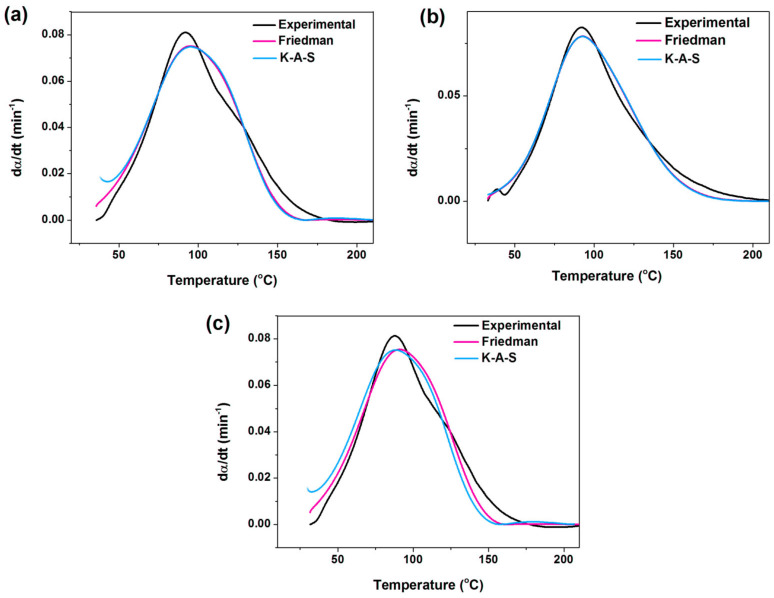
Typical of comparison between experimental and isoconversional approaches at heating rate of 5 °C min^−1^ for EP (**a**), EP/Mica (**b**), and EP/Mica-IM (**c**) systems.

By using the Equations (S6) and (S7) and Figure S4 available in Supplementary Materials, the degrees of autocatalytic (*n*) and non-autocatalytic (*m*) reaction, together with frequency factor (*A*) were calculated, derived from *Friedman* and *KAS* methods (Table 2). The result (*m* + *n*), represents an overall order of network formation. From Table 2, the values of (*m* + *n*) are more than one, indicating the complexity of curing reaction in the epoxy/amine systems. This is the characteristic of the autocatalytic nature of reaction taking place between the epoxy resin and amine curing agent. Furthermore, the autocatalytic reaction order (*m*) increased with incorporation of Mica and Mica-IM into epoxy resin, notifying the improved contribution of autocatalytic reaction to crosslinking. Figure 10 proves how closely the *Friedman* and *KAS* approaches fitted the experimental data on d*α*/d*T* per temperature.

### 3.3. Surface Free Energy Analysis

Table 3 shows the calculated values of the surface free energy (according to the procedure in Section S3 in Supplementary Materials) for the EP, EP/Mica and EP/Mica-IM systems. The non-dispersive (polar) component and dispersive (non-polar) component of the surface energy are also calculated. After Mica was added to the epoxy, the resin became more polar, and even more so for the Mica-IM, although this amount for the EP/Mica-IM has not considerably changed in comparison with the neat epoxy. The high surface energy in the EP/Mica can be attributed to the large hydrogen-bonding ability caused by the incomplete epoxy network formation. On the other hand, the strong interactions in EP/Mica-IM system are the result of the reaction of imidazole with the epoxy, and thereby the surface free energy has decreased.

### 3.4. Viscoelastic Behavior Analysis

**Figure 11:**
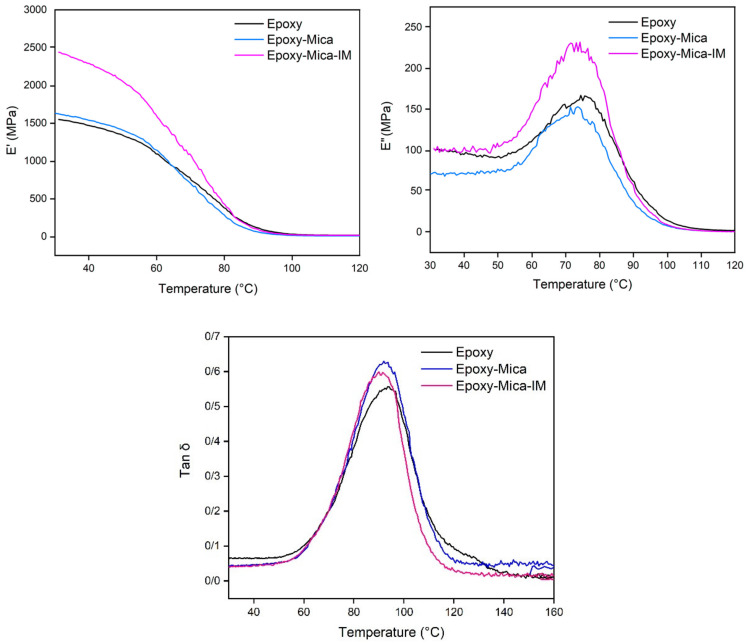
The viscoelastic parameters (E′, E′′ and Tan δ) in terms of temperature at 1 Hz for the studied samples.

Figure 11 shows the results on viscoelastic behavior of blank epoxy and nanocomposites of 0.1 wt % nanoparticles in terms of temperature at 1 Hz frequency, which include storage modulus (E′), loss modulus (E′′) and damping factor (Tan δ). Overall, incorporation of minerals into resins increases the modulus [67,68]. As in Table 4, an expected behavior can be seen, where nanocomposites show higher E′ in the vicinity of glassy region than that of neat resin. In this regard, adding Mica to the epoxy matrix increased the E′ under the glass transition region. This increase is higher for EP/Mica-IM due to the existence of the ionic liquid modified mineral nanoparticles into the coating, which causes the immobility of polymer chain because of the possible interaction between imidazole and epoxy functional groups. Likewise, higher E′ of nanocomposites in the rubbery region suggests intensified crosslinking of epoxy resin in the presence of Mica-IM.

The peaks in Tan δ curve are indicative of *T_g_* (Table 4). *T_g_* values obtained from DSC method at *β* of 10 °C min^−1^ suggest a completely crosslinked network formation. The values *T_g_* for nanocomposites containing Mica and Mica-IM are obviously lower than that of the blank epoxy, which can be attributed to constrained segmental mobility [69]. Nevertheless, *T_g_* of epoxy was limitedly decreased by Mica and Mica-IM incorporation, possibly due to the presence of a few unreacted epoxy resin (less than 5%), a plasticizing effect induced crosslinking [70]. For EP/Mica-IM system, imidazole groups were the reason.

### 3.5. Degradation Kinetics Analysis

**Figure 12:**
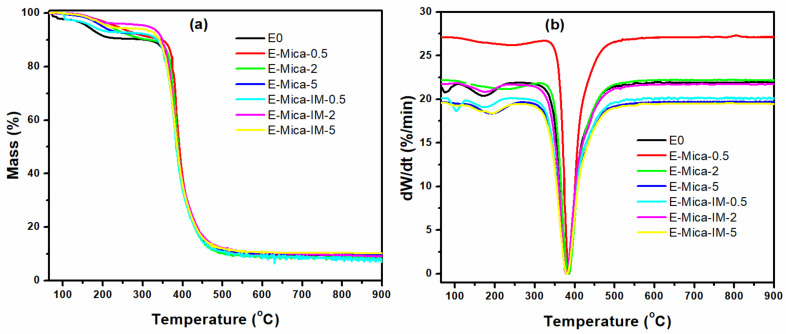
TGA (**a**) and DTG (**b**) thermograms of studied samples.

Figure 12 shows TGA and DTG curves of samples studied in this work. Nanocomposites filled with 0.5, 2, and 5 wt % Mica or Mica-IM underwent thermal decomposition to take their degradation signature at temperature at which 5% mass loss occurs (*T_5_*) and also the peak temperature (*T_P_*) (Table 5). The amount of char residue is also reported in Table 5. As can be seen, all samples have the same decomposition profile and the degradation of the nanocomposites occurs in two steps. The first weight loss of the sample at 120 to 200 °C is probably due to the release of physio-adsorbed water and solvents, and the second and highest weight loss for all samples occurs at 350 to 450 °C, the chief decomposition zone.

We can obviously see from Table 5 that T_5_ is increased for nanocomposites as a whole, which was expected. The value of *T_5_* is higher for EP/Mica-IM compared to the EP/Mica because of the stronger interaction of imidazole groups of Mica-IM with epoxy rings of resin. Furthermore, T_5_ in the high proportion of nanofillers with 5% showed less thermal resistance than that with amounts of 0.5 and 2 wt % due to the high proportion of nanoparticles preventing complete curing of the coating and thus reducing the density of the cross-linking reaction. Accordingly, the samples containing mica-IM 0.5 and 2 wt % have the highest *T_5_*, respectively, which means the presence of modified nanofillers facilitated complete curing and enhance crosslinking density. As a result, the thermal resistance of the coating is improved. Nevertheless, *T_p_* remained stable with negligible change for all samples compared to blank resin.

For kinetics analyses, the conversion of thermal decomposition reaction (*α*) was calculated as [71]:

(8) $$\alpha =\frac{\left(W_{0}-W_{t}\right)}{\left(W_{0}-W_{f}\right)}$$

In the above equation, *W*_0_, *W_t_* and *W_f_* stand for the mass of sample before TGA test, the mass of polymer at time t of experiment, and final mass of the polymer remained at the end of thermal degradation.

**Figure 13:**
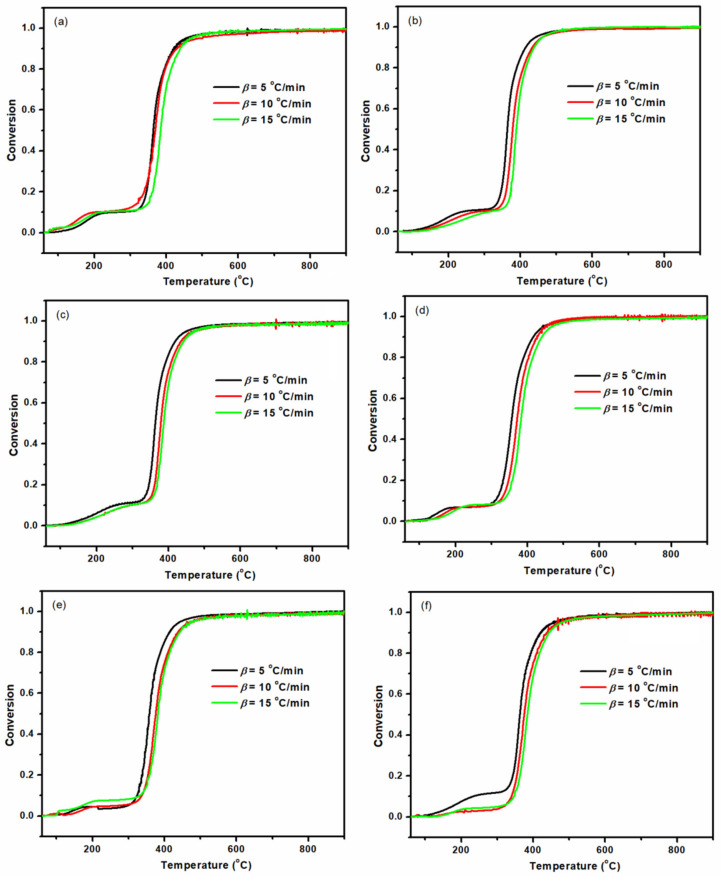
The variation of *α* in terms of temperature for the studied samples at varied *β*, EP/Mica–0.5 (**a**), EP/Mica–2 (**b**), EP/Mica–5 (**c**), EP/Mica–IM–0.5 (**d**), EP/Mica–IM–2 (**e**), and EP/Mica–IM–5 (**f**) samples.

The *α* variation for the studied samples (epoxy nanocomposites containing 0.5, 2 and 5 wt % of Mica or Mica-IM) are obtained as a function of temperature, as shown in Figure 13. Once *β* increased, degradation took place at higher temperatures in correspondence with the increase of the *α* value. It means that a faster pyrolysis would be expectable in a shorter time available to the samples [9].

**Figure 14:**
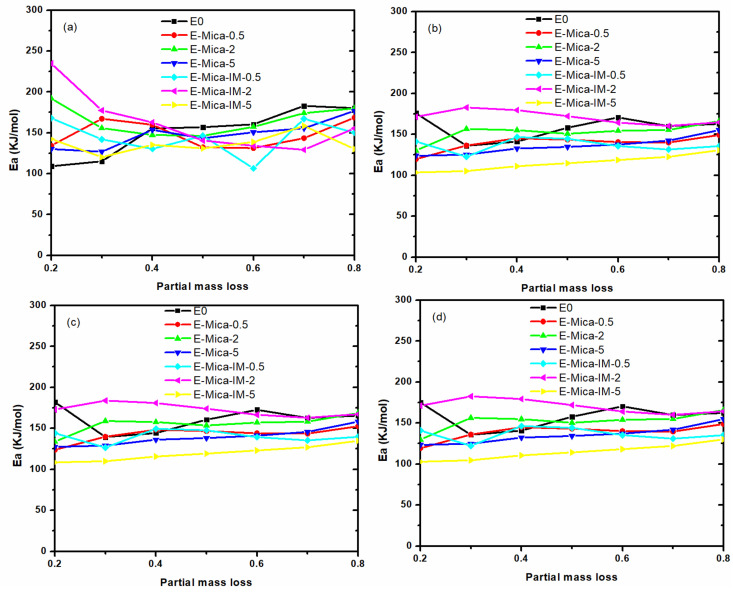
The evolution of the activation energy of thermal degradation against α applying Friedman (**a**) FWO (**b**), KAS (**c**) and m-CR methods (**d**).

Similar to studying the cure kinetics (network formation analysis), we used isoconversional approaches of *Friedman*, Flynn–Wall–Ozawa (*FWO*), *KAS* and modified Coats–Redfern (*m-CR*) to take the signature of the network degradation of the systems in terms of *E_α_* (according to the procedure in Section S4 in Supplementary Materials). Figure 14 compares *E_α_* of degradation reaction (for calculation see Section S4 in SI). Again, the *Friedman* approach usage ended in noisy trend in *E_α_* [72]. On the other hand, using other integral-based isoconversional approaches resulted in well behavior in *E_α_* variation, starting with a descending trend until reaching a plateau. Below *α* value of around 0.4, *E_α_* value of nanocomposites containing 2 wt % nanoparticle (either Mica or Mica-IM) revealed large difference with that of the blank epoxy, which is characteristic of hindered network degradation. The highest *E_α_* value of *E_α_* was obtained for EP/Mica-IM-2 due to the presence of well-dispersed thermally stable Mica nanoplates with imidazolium-based IL on their surface. The imidazole of IL on the surface of Mica can interact with epoxy resin and results in denser network which makes the decomposition reaction more difficult and leads to higher activation energy.

Sestak and Berggren proposed a semi-empirical kinetics model that was used for finding the *f*(*α*) function, which is defined as follows:

(9) $$f(\alpha )=\alpha^{m}\;{\left(1-\alpha \right)}^{n}$$

Table 6 compares parameters of network degradation kinetics for the studied samples. We considered a combination of autocatalytic (by order of *m*) and non-catalytic (by order of *n*) reactions contributing to thermal degradation of the networks. The overall degree of degradation reaction was taken as (*m* + *n*), which increased for epoxy after nanoparticles were added.

**Figure 15:**
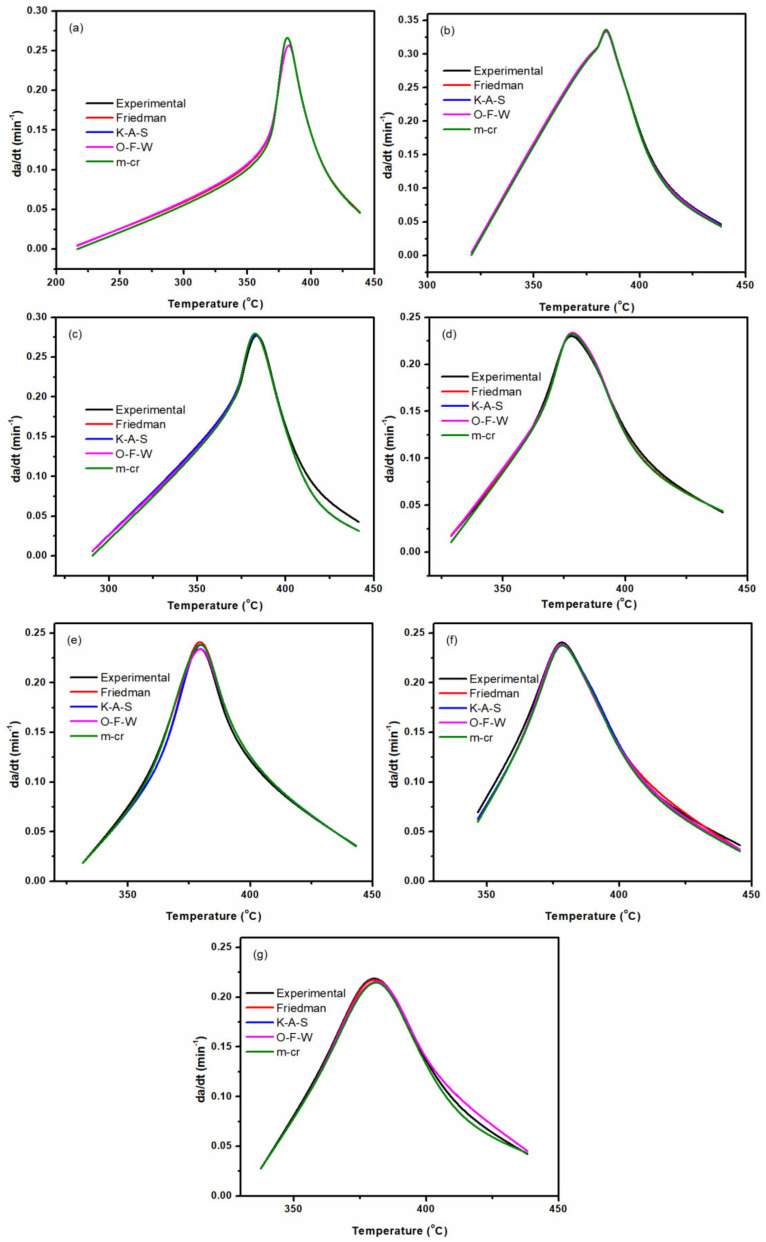
Typical comparison between the experimental and isoconversional approaches at a heating rate of 5 °C min^−1^ for samples E0 (**a**), EP/Mica–0.5 (**b**), EP/Mica–2 (**c**), EP/Mica–5 (**d**), EP/Mica–IM–0.5 (**e**), EP/Mica–IM–2 (**f**), and EP/Mica–IM–5 (**g**) samples.

Figure 15 compares experimental data with values predicted by the isoconversional approaches, where a quite good agreement can be observed.

## 4. Conclusions

Mica surface modified with imidazoline ionic liquid (Mica-IM) was analyzed by the use of XRD and TGA techniques. Then, epoxy nanocomposites with 0.1 wt % Mica or Mica-IM were prepared and analyzed nonisothermally by DSC to investigate the qualitative and quantitative cure parameters of the systems. The *Poor* cure state was identified in EP/Mica and EP/Mica-IM at low *β* of 5 °C min^−1^ caused by hindrance of reaction arising from incorporation of the Mica or Mica-IM. By contrast, the *Good* cure state was obtained at higher *β*, demonstrating the facilitation of crosslinking, as detected by the higher Δ*H* values assigned to the system comprising Mica-IM. The *E_α_* values obtained by *Freidman* and *KAS* isoconversional approaches were obviously lower for nanocomposites compared to the blank epoxy. Thus, network formation was supported by nanoparticle incorporation, particularly in the case of Mica-IM. On the other hand, a higher thermal stability was reflected in the kinetics analysis of network degradation based on *Friedman*, *FWO*, *KAS* and *m-CR* isoconversional methods. In this case, the higher *E_α_* values for the nanocomposites indicated the formation of a denser network after nanoparticle incorporation into the epoxy resin. The TEM micrographs witnessed the obvious formation of the Mica-IM particles with smaller sizes compared to the Mica within the epoxy matrix, which can be considered as a clue for the interpretation of higher properties of Mica-IM based nanocomposites.

## Figures and Tables

**Table 1:** Cure parameters assigned to the blank epoxy and its nanocomposites in terms of heating rate. *T_Onset_* and *T_Endset_* are representative of the onset and endset cure temperatures, and *T_p_* features the peak temperature in the DSC thermograms. In addition, Δ*H_∞_* is the total heat of cure reaction. Red, blue and green colors are representative *Poor*, *Good* and *Excellent* cure states, respectively.

System	Heating Rate (°C min^−1^)	*T_Onset_* (°C)	*T_p_* (°C)	*T_Endset_* (°C)	Δ*T* (°C)	Δ*H_∞_* (J/g)	ΔT*	ΔH*	*CI*	Cure State
**Ep**	5	33.00	91.84	220.00	187.00	329.97	n.a	n.a	n.a	* n.a
	10	37.66	105.86	220.66	183.00	350.67	n.a	n.a	n.a	n.a
	15	42.30	114.33	220.30	178.00	348.44	n.a	n.a	n.a	n.a
**Ep/Mica**	5	35.71	91.98	220.71	185.00	289.82	0.99	0.88	0.87	***Poor***
	10	36.14	106.18	220.14	184.00	400.58	1.01	1.14	1.15	***Good***
	15	33.80	114.25	220.80	187.00	389.02	1.05	1.12	1.17	***Good***
**Epoxy/Mica-IM**	5	31.61	87.85	219.61	188.00	288.53	1.01	0.87	0.88	***Poor***
	10	29.65	99.70	219.65	190.00	405.35	1.04	1.16	1.20	***Good***
	15	34.42	115.31	219.42	185.00	358.65	1.04	1.03	1.07	***Good***

* n.a means not available, for the reference sample.

**Table 2:** Kinetic parameters of the studied samples obtained using *Friedman* and *KAS* isoconversional approaches.

Designation	Heating Rate (°C/min)	*Friedman*			*KAS*		
		*m*	*n*	ln*A* (s^−1^)	*m*	*n*	ln*A* (s^−1^)
**Epoxy**	5	0.12	2.04	17.74	0.00	2.24	21.65
	10	0.15	2.03	17.74	0.01	2.23	21.50
	15	0.15	1.94	17.69	0.01	2.12	21.37
**Epoxy/Mica**	5	0.18	1.55	15.22	0.20	1.78	20.68
	10	0.23	1.93	15.68	0.1	2.20	20.97
	15	0.23	1.86	15.71	0.01	2.11	20.87
**Epoxy/Mica-IM**	5	0.34	1.51	14.51	0.13	1.68	18.50
	10	0.24	1.95	15.09	0.08	2.15	18.98
	15	0.31	1.68	14.93	0.14	1.83	18.66

**Table 3:** The theoretically calculated values of surface free energy of the studied samples.

Samples	Dispersive Component	Polar Component	Total Energy
**EP**	34.2	2.8	37.0
**EP/Mica**	27.6	10.3	37.9
**EP/Mica-IM**	35.7	1.3	37.0

**Table 4:** Values of T_g_ of completely cured samples collected at β of 10 °C min^−1^.

Sample	*T_g_* (°C)		Storage Modulus (MPa)	
	DMA	DSC	Glassy Region	Rubbery REGION
**EP**	94.11	94.98	1572	9.5
**EP/Mica**	93.64	89.02	1657	11.9
**EP/Mica-IM**	91.90	90.99	2522	19.8

**Table 5:** Parameters of TGA test for the studied samples.

Sample	*T_5_* (°C)	*T_P_* (°C)	Residue (%) at 900 °C
**EP**	147.59	369.91	12.24
**EP/Mica-0.5**	207.10	376.36	11.20
**EP/Mica-2**	221.49	376.01	9.70
**EP/Mica-5**	184.14	369.19	8.20
**EP/Mica-IM-0.5**	282.97	370.80	5.99
**EP/Mica-IM-2**	320.02	370.83	7.23
**EP/Mica-IM-5**	179.86	364.90	7.30

**Table 6:** Parameters of degradation kinetics obtained for the studied samples.

	*Friedman*			*FWO*			*KAS*			*m-CR*		
Designation	Ln *A* (min^−1^)	*m*	*n*	Ln *A* (min^−1^)	*m*	*n*	Ln *A* (min^−1^)	*m*	*n*	Ln *A* (min^−1^)	*m*	*n*
E0	16.1	1.8	1.5	20.7	0.7	1.2	21.4	0.8	1.5	20.6	0.4	0.7
EP/Mica-0.5	27.3	0.2	2.3	23.9	0.3	2.3	24.5	0.1	2.3	23.7	0.2	1.2
EP/Mica-2	27.0	0.6	2.1	25.7	0.6	2.6	26.2	0.5	2.3	25.6	0.3	0.6
EP/Mica-5	27.5	0.7	3.2	26.8	0.9	2.7	27.4	0.7	3.3	26.7	0.7	2.3
EP/Mica-IM-0.5	21.3	1.1	2.6	26.6	1.1	2.6	27.2	0.8	3.0	26.5	0.8	2.0
EP/Mica-IM-2	26.0	1.5	1.5	26.5	1.6	1.6	26.9	1.6	1.3	26.9	1.3	0.5
EP/Mica-IM-5	17.8	1.8	2.5	16.1	1.6	2.4	16.8	1.9	2.4	15.7	1.6	2.4

## Supplementary Materials

The following are available online at https://www.mdpi.com/article/10.3390/nano11081990/s1; Figure S1. Isoconversional plots of prepared samples based on (a) Friedman and (b) KAS models, Figure S2. Plots of ln [Af(α)] vs. ln(1-α) for (a) EP, (b) EP/Mica and (c) EP/Mica-IM nanocomposites under heating rate of 5 °C.min^–1^ via Friedman method, Figure S3. Variation of y(α) and Z(α) versus conversion for (a) EP (b) EP/Mica and (c) EP/Mica-IM nanocomposites based on Malek model at heating rate of 5 °C min^–1^, Figure S4. Plots of (a) Value I and (b) Value II calculated using DSC data for EP, EP/Mica and EP/Mica-IM nanocompo-sites under heating rate of 5 °C.min^–1^.
