# Supplementary material for: Epoxy/Ionic Liquid-Modified Mica Nanocomposites: Network Formation–Network Degradation Correlation
Source: Nanomaterials (Basel). 2021 Aug 3;11(8):1990. doi: 10.3390/nano11081990 (PMC8400988; doi:10.3390/nano11081990)
Supplement: Supplementary file 1 [file nanomaterials-11-01990-s001.zip › nanomaterials-1286354-supplementary.pdf]

nanomaterials

Supplementary Materials

Epoxy/Ionic Liquid-Modified Mica Nanocomposites: Network Formation–Network Degradation Correlation

Maryam Jouyandeh [1], Vahideh Akbari [1], Seyed Mohammad Reza Paran [2], Sébastien Livi [3], Luanda Lins [4], Henri Vahabi [1,*] and Mohammad Reza Saeb [5,*]

[1] Université de Lorraine, CentraleSupélec, LMOPS, F-57000 Metz, France; maryam.jouyande@gmail.com (M.J.); vahidehakbari1991@gmail.com (V.A.)
[2] Center of Excellence in Electrochemistry, School of Chemistry, College of Science, University of Tehran, 1417935840 Tehran, Iran, smrparan@gmail.com
[3] Université de Lyon, CNRS, UMR 5223, Ingénierie des Matériaux Polymères, INSA Lyon, F-69621 Villeurbanne, France; sebastien.livi@insa-lyon.fr
[4] Université de Lyon, CNRS, UMR 5510, MATEIS, INSA Lyon, F-69621 Villeurbanne, France; luandaqmc@gmail.com
[5] Department of Polymer Technology, Faculty of Chemistry, Gdańsk University of Technology, G. Narutowicza 11/12, 80-233 Gdańsk, Poland
* Correspondence: henri.vahabi@univ-lorraine.fr (H.V.); mrsaeb2008@gmail.com (M.R.S.); Tel.: +33(0)3-87-93-91-86 (H.V.); +989128264307 (M.R.S.)

S1. Materials and Methods

Epoxy resin and its curing agent were Renlam CY219 (viscosity of 10’000-12’000 mPas at 25 ºC and density of 1.1 g/cm^3) and REN HY 5161 (viscosity of 35–55 mPas at 25 ºC and density of 1.0 g/cm^3), respectively, provided by Huntsman, Germany. All the solvents used in this survey including methyl alcohol, tetrahydrofuran (THF), sodium methanoate, and acetonitrile, and octadecyl iodide (95%), were purchased from Aldrich and used as received based on the recipes. Mica is a synthetic fluorohectorite, denoted Somasif ME-100 and was provided by CO-OP chemical Co., Japan. The Somasif ME–100 has a cation exchange capacity of 70 meq/100g and is described by the following formula $Na_{2x}Mg_{3.0-x}Si_4O_{10}(F_yOH_{1-y})_2$ (x = 0.15–0.5; y = 0.8–1.0).

The synthesis procedure of imidazolium ionic liquid was based on a previous study [1]. In brief, sodium methoxide solution was prepared from 1 equiv. of sodium using freshly distilled methyl alcohol (10 mL) in a sealed septum, 100 mL round-bottom, three-necked flask equipped with a condenser, under argon atmosphere and magnetic stirring. Then, imidazole (1 equiv.) diluted using acetonitrile (10 mL) added into the stirred mixture of sodium methoxide at ambient temperature. The white precipitate was witnessed after 30 min. The suspension was concentrated under reduced pressure for 1 h. The dried white powder was dissolved in acetonitrile. The solution of octadecyl iodide (1 equiv.) diluted in acetonitrile (10 mL) was then added at room temperature and under an inert atmosphere of nitrogen. The mixture was stirred for 1 h and then heated under reflux at 85 ˚C for about 24 h. After twice time of the procedure repetition, the solvent was removed by evaporation under vacuum. Finally, purification was carried out by crystallization in a mixture (75/25) of ethyl acetate/acetonitrile.

According to the previous works, the intercalation of the hydrophilic swellable mica (Somasif ME-100) by organic cations in an aqueous medium was performed [2–4]. The synthetic layered silicates (2 g, 1.9 meq) were dispersed in 400 mL of deionized water with 2 CEC of the imidazolium IL corresponding to the cation exchanged capacity (CEC = 75 meq/100 g) of the synthetic layered silicate used [5]. The mixture was then stirred and mixed at 80 °C during 24 h. After various washing steps with hot water and hot ethanol in order to remove physisorbed ionic liquid, the resulting organically modified mica was dried during one night at 80 °C and stored in a desiccator. The following abbreviation was

used to design the imidazolium-treated mica denoted IM, when N-octadecyl-N′-octadecylimidazolium iodide was used as surfactant agent.

A mixture of epoxy resin, THF and 0.1 wt.% of nanofiller was sonicated (under on/off cycle) for 10 min and was left for 24 h for eliminating air bubbles. Then, hardener in appropriate ratio (the ratio of epoxy to hardener was 2:1 by weight) was added to the epoxy prepolymer to embark curing process, after 3 min a homogeneous mixture was obtained. In order to prevent curing before DSC measurements, nanocomposites were placed under –10 ˚C. The same procedure was used for nanocomposites containing 0.5, 2.0, and 5.0 wt.% Mica and Mica-IM (prepared similar to 0.1 wt.% mixture) used in thermal degradation analysis.

The structure of mica nanoparticles was assessed by X-ray diffraction (XRD) analysis using diffractometer, Rigaku-MiniflexII (Japan). The CuKα radiation was done at current of 15 mA and voltage of 30 kV and 2θ values in the range of 5° to 70°.

A Setaram Labsys Evo thermogravimetric analyzer (France) was used for TGA study for both nanoparticles and nanocomposites. Nanoparticles were characterized at heating rate of 10 °C.min^{-1} in the temperature range of 30–900 °C under a nitrogen purge of 50 mL.min^{-1} inspired by previous attempts [6]. Thermal behavior and kinetics of epoxy nanocomposites in the presence of 0.5, 2.0, and 5.0 wt.% of Mica and Mica-IM were investigated at heating rates of 5, 10 and 15 ˚C.min^{-1}. Transmission electron microscopy (TEM) Phillips CM 120 microscope working at 80 kV in Technical Center of Micro-structures (University of Lyon) was used to probe into the morphology and dispersion state of mica and mica-IM in the epoxy matrix.

Analysis of the cure behavior and kinetics of epoxy nanocomposites was carried out based on nonisothermal DSC measurements by a NETZSCH 200F3 Maia Differential Scanning Calorimeter (Netherland) and a 409 PC Luxx Simultaneous Thermal Analyzer (STA). All DSC analyses were accomplished under nitrogen purging of 50 mL.min^{-1}. Three heating rates (5, 10, and 15 ˚C.min^{-1}) were considered to evaluate the cure reaction and estimation of T_g (at heating rate of 10 ˚C.min^{-1}) by heating the samples from 15 ˚C to 300 ˚C, then cooling down to the room temperature at a constant cooling rate and reheating to 300 ˚C.

The sessile drop method with a Dataphysics goniometer using water and diiodomethane as probe liquids were used to measure surface free energy of fully cured EP, EP/Mica, and EP/Mica-IM nanocomposites. Viscoelastic behavior of nanocomposites was assessed through Dynamic mechanical analysis (DMA) at the frequency of 1 Hz from 20 ˚C up to 200 ˚C with a heating rate of 3 ˚C.min^{-1} and with a 120 mm thickness, 10 mm width, and 50 mm length.

S2. Cure Analysis Details

Differential *Friedman* and *KAS* methods for isoconversional model are described as Equation (S1) and Equation (S2), respectively:

$$ln\left[\beta_i\left(\frac{d\alpha}{dT}\right)_{\alpha,i}\right] = ln\left[f(\alpha)A_\alpha\right] - \frac{E_\alpha}{RT_{\alpha,i}} \quad \text{(S1)}$$

$$ln\left(\frac{\beta_i}{T_{\alpha,i}^{1.92}}\right) = Const - 1.0008\left(\frac{E_\alpha}{RT_\alpha}\right) \quad \text{(S2)}$$

Figure S1 (a) is demonstrated that in each α the value of E_α can evaluate from the slope of the curve of $ln\left[\beta_i\left(d\alpha/dT\right)_{\alpha,i}\right]$ vs. $1/T_\alpha$.

In addition, the activation energy based on *KAS* method is calculated from slope of $ln\left(\beta_i/T_{\alpha,i}^{1.92}\right)$ vs. $1/T$ (Figure S1 (b)).

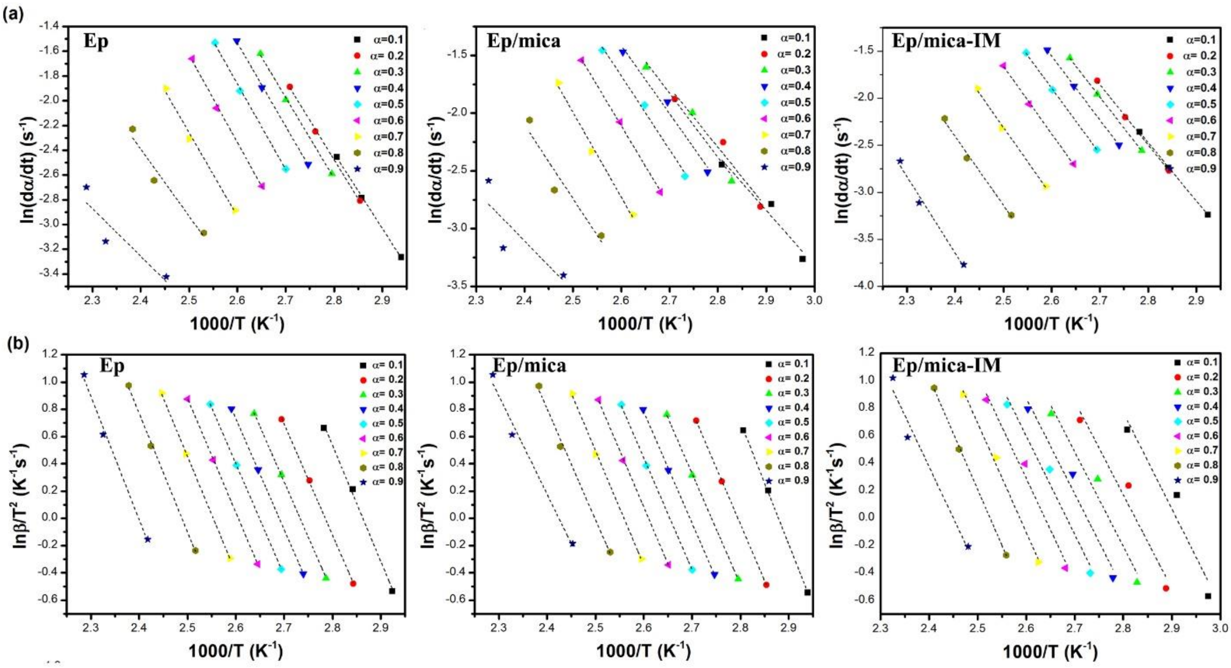

Figure S1. Isoconversional plots of prepared samples based on (**a**) Friedman and (**b**) KAS models.

Equation (S3) is presented to find out the model of curing reaction based on *Freidman* model. The figure of plot of ln[$Af(\alpha)$] vs. ln(1–α) defines the deviation from nth order reaction (Figure 9) [7].

$$ln\left[Af(\alpha)\right] = ln\left(\frac{d\alpha}{dt}\right) + \frac{E}{RT} = lnA + nln\left(1-\alpha\right) \quad \text{(S3)}$$

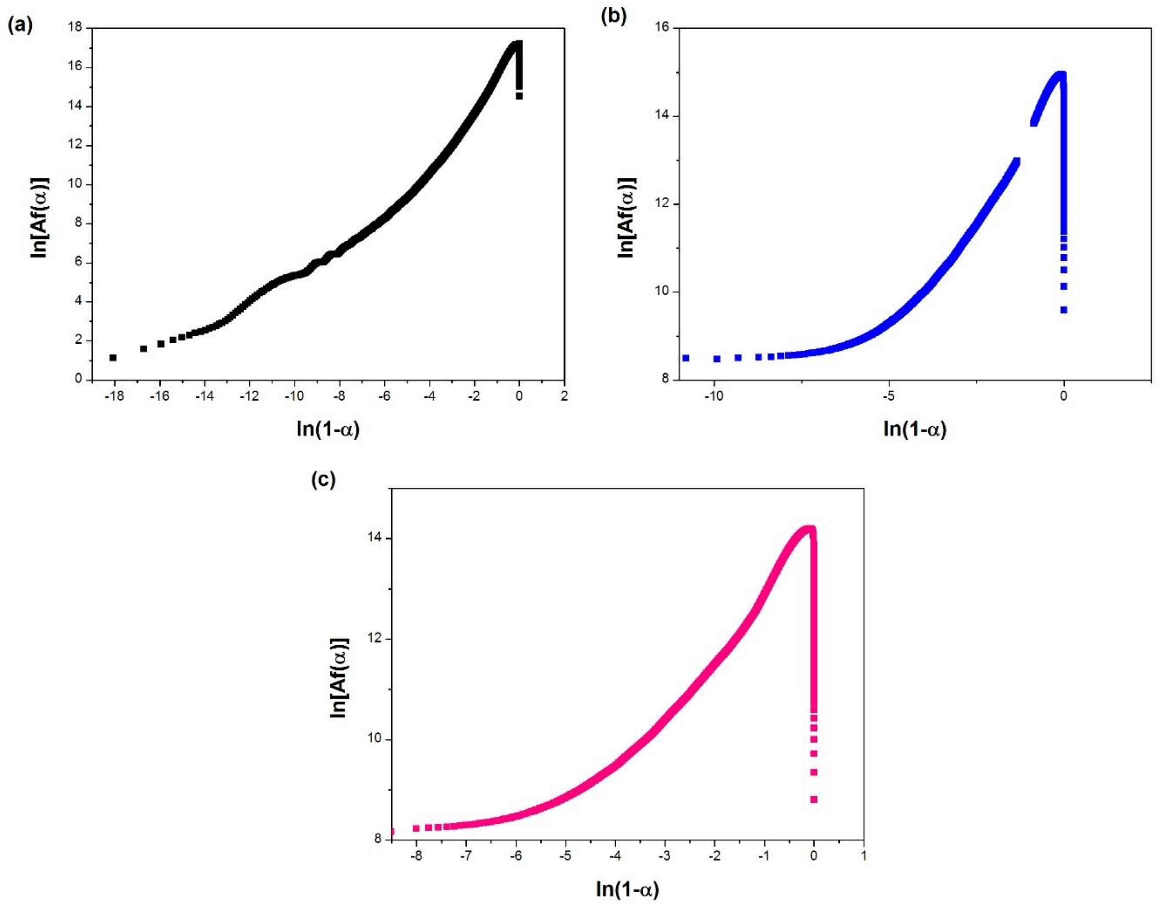

Figure S2. Plots of ln [$Af(\alpha)$] vs. ln(1–α) for (**a**) EP, (**b**) EP/Mica and (**c**) EP/Mica-IM nanocomposites under heating rate of 5 °C.min^{-1} via Friedman method.

The following equations can be used to distinguish the kinetic model derived using the *Malek* method:

$$y(\alpha) = \left(\frac{d\alpha}{dt}\right)_\alpha exp\left(\frac{E_0}{RT_\alpha}\right) = Af(\alpha) \quad \text{(S4)}$$

$$z(\alpha) = \left(\frac{d\alpha}{dt}\right)_\alpha T_\alpha^2 \quad \text{(S5)}$$

The theoretical master plots of y(α) and z(α) for EP, EP/Mica and EP/Mica-IM nanocomposite as a function of conversion and experimental values are compared in Figure S3.

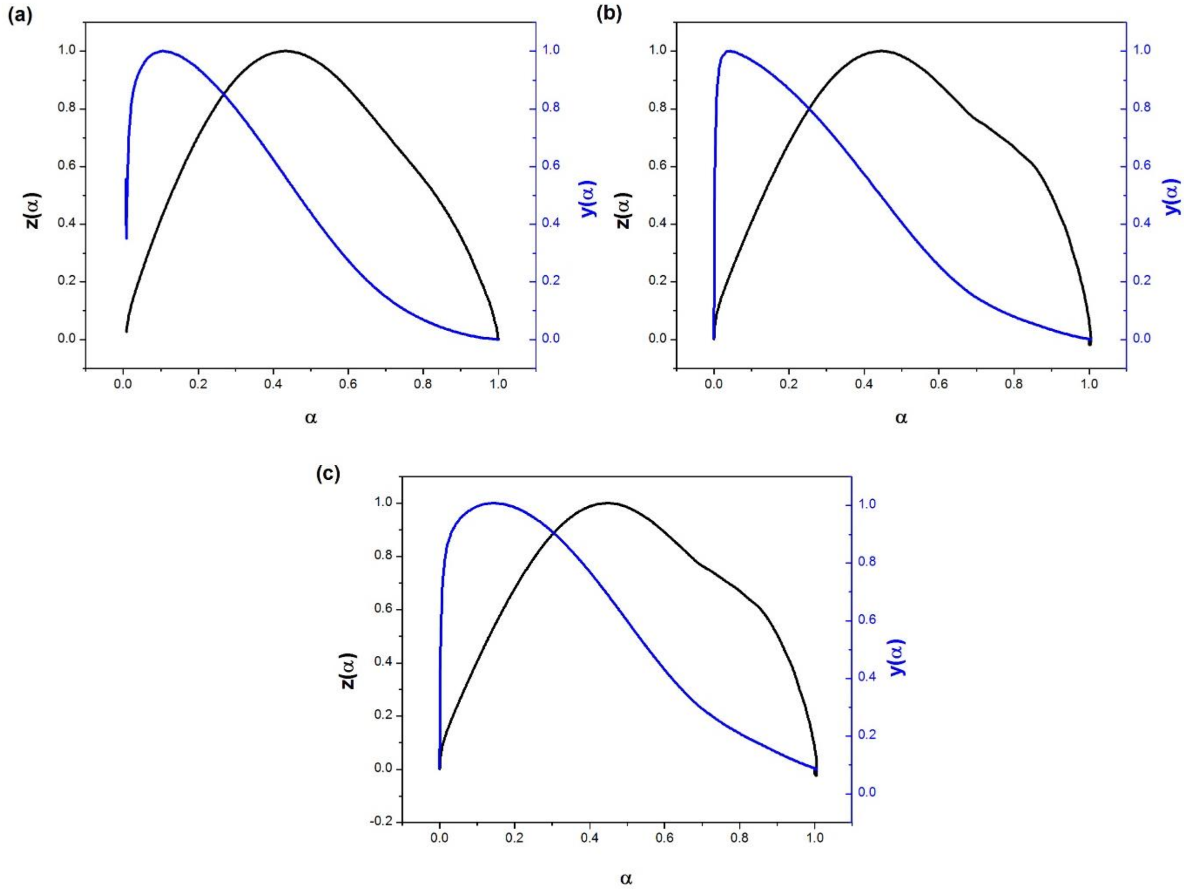

Figure S3. Variation of $y(\alpha)$ and $Z(\alpha)$ versus conversion for (**a**) EP (**b**) EP/Mica and (**c**) EP/Mica-IM nanocomposites based on *Malek* model at heating rate of 5 °C min^{-1}.

By using the Equation (S6) and Equation (S7), the degrees of autocatalytic reaction (n and m) and the pre-exponential factor (A) can be calculated.

$$ValueI = ln\left(\frac{d\alpha}{dt}\right) + \frac{E_\alpha}{RT} - ln\left[\frac{d(1-\alpha)}{dt}\right] - \frac{E_\alpha}{RT'} = (n-m)ln\left(\frac{1-\alpha}{\alpha}\right) \qquad (S6)$$

$$ValueII = ln\left(\frac{d\alpha}{dt}\right) + \frac{E_\alpha}{RT} + ln\left[\frac{d(1-\alpha)}{dt}\right] + \frac{E_\alpha}{RT'} = (n+m)ln(\alpha-\alpha^2) + 2lnA \qquad (S7)$$

According to the Figure S4 (a) and Figure S4 (b), the slope of the plot of ValueI vs. ln $[(1-\alpha)/\alpha]$ and ValueII vs. $\ln(\alpha-\alpha^2)$ give the value of $(n-m)$ and the value of $(n+m)$ and $2\ln A$, respectively.

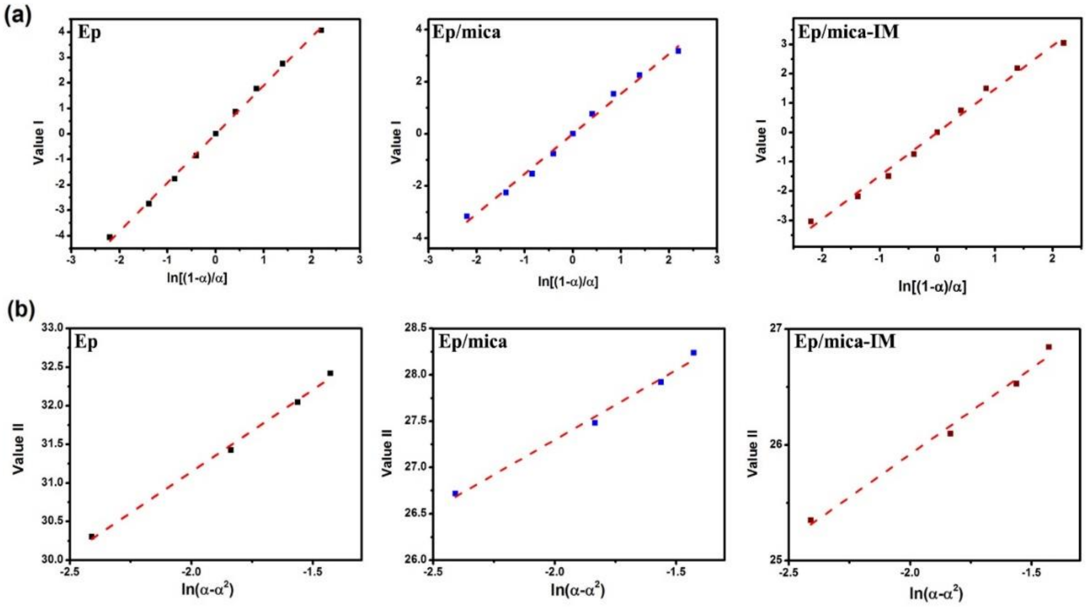

Figure S4. Plots of (**a**) *Value* I and (**b**) *Value* II calculated using DSC data for EP, EP/Mica and EP/Mica-IM nanocomposites under heating rate of 5 °C.min^{-1}.

S3. Calculation of Surface Energy

The total surface free energy is divided into two components of non-dispersive and dispersive components.

$$\gamma=\gamma^{L}+\gamma^{SP} \quad (S8)$$

where γ^{L} is the London dispersive component and γ^{SP} is the specific (or polar) component. γ^{L} indicates Van der Waals interactions which encompass London dispersion forces and γ^{SP} associates with Debye-inductive polarization, Keesom-orientational polarization forces, and hydrogen bonding [65].

Non-dispersive (polar and H-bond) and dispersive components of surface energy were cal-culated using the Owens-Wendt theory [66].

The term γ^{SP} is further divided into two parameters using geometric mean:

$$\gamma^{SP}=2\sqrt{\gamma^{+}\gamma^{-}} \quad (S9)$$

where γ^{+} and γ^{-} are the electron-acceptor and the electron-donor parameters of the specific polar component of surface free energy, respectively.

The Young equation which shows the contact angle of a liquid on a solid in terms of the dispersion force contributions of each is defined as follows:

$$\gamma_{L}\cos\theta=\gamma_{S}-\gamma_{SL} \quad (S10)$$

where γ_{L}, γ_{S} and γ_{SL} refer to free energies of the liquid and solid and their interfacial tension, respectively. γ_{SL} can be calculated from the following expression:

$$\cos\theta=-\gamma_{SL}=\gamma_{S}+\gamma_{L}-2\sqrt{\gamma_{S}^{L}\gamma_{L}^{L}}-2\sqrt{\gamma_{S}^{SP}\gamma_{L}^{SP}} \quad (S11)$$

Therefore, Equation (S11) can be rewritten as:

$$1+\cos\theta=2\sqrt{\gamma_{S}^{L}}\left(\frac{\sqrt{\gamma_{L}^{L}}}{\gamma_{L}}\right)+2\sqrt{\gamma_{S}^{SP}}\left(\frac{\sqrt{\gamma_{L}^{SP}}}{\gamma_{L}}\right) \quad (S12)$$

By measuring θ for two liquids of water and diiodomethane and considering their known values of γ_{L}^{L}, γ_{L}^{+} and γ_{L}^{-} a set of equations can be simultaneously solved to obtain the surface free energy for the solid.

S4. Thermal Degradation Calculation

Flynn–Wall–Ozawa (*FWO*) is defined as the following equation [8]:

$$ln(\beta_i) = Const - 1.052\left(\frac{E_\alpha}{RT_\alpha}\right) \quad \text{(S13)}$$

The *m-CR* method is defined as [67]:

$$ln\left[\frac{\beta}{T^2(1-2RT/E_\alpha)}\right] = ln\left[\frac{-AR}{E_\alpha ln(1-\alpha)}\right] - \frac{E_\alpha}{RT} \quad \text{(S14)}$$

By plotting $ln\left[\beta/T^2(1-2RT/E_\alpha)\right]$ against $1/T$, a straight line was obtained, which slope gives the activation energy of degradation reaction. The $E\alpha$ values from *Friedman* and *KAS* methods can be obtained from Equation (S1 and S2).

References

1. Nguyen, T.K.L. New generation of epoxy networks based on ionic liquids: From structuration to final properties. Université de Lyon, 2016.
2. Livi, S.; Duchet-Rumeau, J.; Pham, T.-N.; Gérard, J.-F. A comparative study on different ionic liquids used as surfactants: Effect on thermal and mechanical properties of high-density polyethylene nanocomposites. *Journal of colloid and interface science* **2010**, *349*, 424–433.
3. Livi, S.; Duchet-Rumeau, J.; Gérard, J.F. Effect of ionic liquid modified synthetic layered silicates on thermal and mechanical properties of high density polyethylene nanocomposites. In Proceedings of the Macromolecular Symposia, 2014; pp. 46–55.
4. Livi, S.; Sar, G.; Bugatti, V.; Espuche, E.; Duchet-Rumeau, J. Synthesis and physical properties of new layered silicates based on ionic liquids: improvement of thermal stability, mechanical behaviour and water permeability of PBAT nanocomposites. *RSC Adv.* **2014**, *4*, 26452–26461.
5. Lee, S.Y.; Cho, W.J.; Kim, K.J.; Ahn, J.H.; Lee, M. Interaction between cationic surfactants and montmorillonites under nonequilibrium condition. *Journal of colloid and interface science* **2005**, *284*, 667–673.
6. Seidi, F.; Jouyandeh, M.; Paran, S.M.R.; Esmaeili, A.; Karami, Z.; Livi, S.; Habibzadeh, S.; Vahabi, H.; Ganjali, M.R.; Saeb, M.R. Imidazole-functionalized nitrogen-rich Mg-Al-CO3 layered double hydroxide for developing highly crosslinkable epoxy with high thermal and mechanical properties. *Colloids and Surfaces A: Physicochemical and Engineering Aspects* **2021**, *611*, 125826, doi:https://doi.org/10.1016/j.colsurfa.2020.125826.
7. Sbirrazzuoli, N. Is the Friedman method applicable to transformations with temperature dependent reaction heat? *Macromolecular Chemistry and Physics* **2007**, *208*, 1592–1597.
8. Ozawa, M.; Kimura, M.; Isogai, A. Thermal stability and characterization of γ-Al2O3 modified with rare earths. *Journal of the Less Common Metals* **1990**, *162*, 297–308.
